# Dynamic grasping system based on visual algorithm and robot arm collaboration in logistics production line

**DOI:** 10.1371/journal.pone.0340455

**Published:** 2026-01-09

**Authors:** Bowen He, Bin Chen

**Affiliations:** Mechanical and Electrical Engineering College, Hainan Vocational University of Science and Technology, Haikou, China; King Fahd University of Petroleum & Minerals, SAUDI ARABIA

## Abstract

In response to the urgent need for efficient and accurate dynamic grasping in automated logistics, this study proposes the VRCDS, a dynamic grasping system that integrates a multi-feature weighted PnP vision algorithm with multi-robot arm collaboration. The system establishes a closed-loop “perception-decision-execution-feedback” architecture, significantly enhancing grasping accuracy, system efficiency, and energy savings compared to traditional single-arm or visual servo schemes. Experimental results demonstrate that the VRCDS system achieves high recognition accuracy, robust grasping efficiency under various conveyor speeds, precise trajectory control, and a substantial reduction in power consumption. The research provides an efficient, precise, and reliable solution for dynamic grasping tasks in logistics automation.

## 1. Introduction

As an important pillar of the national economy, the automation level of the logistics industry has a far-reaching impact on the efficiency of social production and the speed of commodity circulation [1]. Currently, the rapid growth of e-commerce has led to an increasingly diverse range of consumer demands. This has created unprecedented challenges and opportunities for logistics production lines [2]. Traditional manual sorting and handling methods can no longer meet the needs of modern logistics for high efficiency, high precision and low cost. Especially in large-scale and high-complexity logistics tasks, the error rate and time cost of manual operation have increased significantly, seriously restricting the overall efficiency of the logistics industry. To improve the efficiency and accuracy of logistics sorting, handling and packaging, and to reduce the labor cost and error rate, a dynamic gripping system has become a research hotspot in the field of logistics automation [3,4]. Existing research mostly focuses on the grasping operation of a single mechanical arm, and lacks an in-depth exploration of the collaboration mechanism of multiple mechanical arms. As a result, large-scale and complex logistics tasks limit overall system efficiency and flexibility [5]. To this end, the study proposes a dynamic grasping system based on the collaboration of vision algorithm and mechanical arm. By fusing multi-feature weighted perspective-n-point (PnP), strengthening feature robustness, and introducing a dynamic error compensation mechanism, the system significantly improves the positioning accuracy and grasping stability of the target object. Meanwhile, the introduction of mechanical arm collaboration mechanism realizes the cooperative work of multiple mechanical arms in the logistics production line, which improves the adaptability and execution efficiency of the system to complex tasks. Finally, the system is automated based on programmable logic controller (PLC), which further optimizes the operational efficiency and stability of the system and achieves efficient and accurate control of the mechanical arm gripping motion. The innovation of the research is to propose a dynamic grasping framework that integrates multi-feature weighted PnP algorithm and mechanical arm collaboration, which significantly reduces the grasping error. A hierarchical collaborative control strategy is designed to achieve the optimization of task allocation and path planning for multiple mechanical arms. The efficiency and stability of the system in real logistics scenarios are verified through experiments. The research aims to improve the efficiency and accuracy of logistics sorting and handling, reduce the labor cost and error rate, and provide new solutions for the intelligent development of logistics industry.

This work fundamentally differs from prior research by proposing a system-level closed-loop architecture, rather than making incremental improvements to single technologies. It integrates a multi-feature weighted PnP visual algorithm, hierarchical multi-arm collaboration, and PLC-based hard real-time execution into a complete “perception-decision-execution-feedback” loop, effectively solving collaboration and robustness challenges in dynamic logistics scenarios.

The innovations of this study are as follows: (1) A novel system-level closed-loop architecture: For the first time, the proposed integrated framework seamlessly connects a robust visual perception module (a multi-feature weighted PnP algorithm), an intelligent multi-arm coordination strategy, and a deterministic PLC-based hard real-time execution layer. This creates a full “perception-decision-execution-feedback” loop that fundamentally overcomes the limitations of collaboration and robustness of loosely integrated systems in dynamic logistics scenarios. (2) A robust visual algorithm with dynamic compensation: The proposed multi-feature weighted PnP algorithm dynamically fuses diverse visual features and incorporates a real-time error compensation mechanism. This design provides high positioning accuracy and resilience against common industrial disturbances, such as lighting variations, occlusions, and vibrations. This is a significant advancement over approaches that depend on a single algorithm or require a lot of data. (3) Deep integration of collaborative planning and execution: Moving beyond algorithmic-level coordination, the proposed hierarchical control strategy is deeply integrated with the PLC control system. This integration bridges the critical gap between high-level collaborative planning and low-level actuation. It enables the deterministic and reliable execution of complex, multi-arm tasks. Thus, it achieves a leap from “collaborative planning” to “collaborative execution”.

## 2. Literature review

A logistics robotic arm is a programmable manipulation device, driven by computer control and sensor feedback, designed for automating material handling tasks. It can simulate the actions of a human arm and perform operations such as grasping, handling, sorting, palletizing, and loading and unloading goods. It is one of the core pieces of equipment in modern intelligent logistics systems. It is one of the core equipment in a modern intelligent logistics system. A large number of scholars have carried out relevant researches on it. Dabic-Miletic S et al. explored the integrated application of AI and robotics in warehouse management systems, aiming to enhance operational efficiency and meet sustainability needs [6]. Ha S H et al. developed a logistics automation system using cable-driven parallel robots to address maintenance issues related to high-level shelving systems [7]. Zhu W et al. developed a practical robotic crating system for the difficult problem of three-dimensional dynamic heterogeneous robot palletizing in logistics and distribution centers. The study ensured mechanical arm collision-free operation and stacking stability by establishing an executability model, and proposed an efficient algorithm to calculate the mechanical arm collision-free trajectory. Experimental results indicated that the designed system could effectively solve the palletizing problem [8]. Zermane A et al. proposed a decoupled two-layer planning control strategy for the complex task requirements of robotic impact maneuvers in logistics sorting scenarios. The study planned mechanical arm non-stop cyclic trajectories in the top layer, while the bottom layer mapped task-space impact targets to joint-space path points via a task-aware model plug-in. Experimental results indicated that the proposed strategy made the robot impact accuracy effectively improved [9]. Vijayakumar V et al. developed an optimization model based on picking robots for e-commerce warehousing to balance system productivity and operational quality [10]. Borangiu T et al. proposed an intelligent palletization model with a holonic and individual paradigm for a conventional centralized system that struggles to cope with order fluctuations and equipment anomalies. The study constructed a two-tier Holonic System logistics execution system that synthesizes robot speed limits and inventory costs. Experimental results indicated that the proposed model could effectively solve abnormal situations [11].

The development of mechanical arm vision algorithms so far, some of the theories and practical applications have been relatively mature. Scholars in many countries have conducted in-depth research on them. In response to the core challenge of the unknown kinematic model of the robotic arm, Yu et al. innovatively proposed a pose control method based on double-gradient neural dynamics. The significant contribution of this research lied in the design of a cerebellar heuristic control loop. By simulating the online learning and coordination functions of the biological cerebellum, it effectively enhanced the pose control accuracy of the end effector. However, in highly dynamic capture scenarios, there might be a discrepancy between the time it takes for the model to converge and the system’s real-time requirements [12]. Ha et al. took a different approach and constructed a visual servoing framework based on self-supervised learning. The core of this framework was its use of a double contrastive learning algorithm to autonomously extract robust features that were insensitive to illumination and occlusion from multi-view images. These features were then ingeniously integrated with the kinematic model of the robotic arm to construct a temporal state prediction network. This end-to-end learning paradigm allowed for outstanding adaptability in unstructured environments. However, performance largely depended on pre-training with a large amount of unlabeled data, and the computational overhead was relatively high [13]. Huang C I et al. proposed a federated hybrid attention network (Fed-HANet) system based on federated learning to address the problem of privacy protection and difficult data acquisition in human-robot handover tasks for service robots. The study adopted a federated learning framework to train an end-to-end control model, and realized unknown object grasping through deep vision with red, green, blue (RGB) input fusion. Experimental results indicated that the proposed system could effectively handover privacy protection and data acquisition in the task [14]. Cong V D et al. proposed an image-based visual servo sorting system to address the lack of flexibility and complex configuration of traditional sorting robot systems. The study used decoupled visual servo control of a 4-degree-of-freedommechanical arm, and extracted the area, azimuth, and center-of-mass features of the target object as control inputs through a multi-threshold algorithm. Experimental results indicated that the system realized real-time image processing on a low-cost embedded computer [15]. Wang et al. focused their work on balancing the accuracy and speed of object detection. The study proposed a visual positioning algorithm based on an anchor-free detection network. This algorithm significantly optimized the computational efficiency of the network’s forward reasoning by using structural re-parameterization technology. Meanwhile, by integrating multi-scale feature fusion with deformable convolutional modules, the model’s ability to recognize multiple targets under scale variations and complex backgrounds was enhanced. This algorithm reliably supported the precise, real-time grasping of robotic arms. However, the performance of its detection could be affected by severely overlapping targets or motion blur [16]. Gkeka et al. addressed the issues of real-time performance and vibration interference in visual positioning from a hardware acceleration perspective. They designed a real-time bitmap optimization system based on a field programmable gate array (FPGA) to achieve a feature processing speed in the microsecond range. They did this by improving the classic ORB feature detection algorithm and deploying it on dedicated hardware. The collaborative design of the software and hardware effectively compensated for the vibration blurring caused by the robotic arm’s high-speed movement. This ensured the stability of the visual positioning system during high-speed operation. However, the system’s flexibility was limited by its specific hardware architecture [17]. Although significant progress has been made in existing research on dynamic logistics, most of the work focuses on progressively optimizing a single technology. This work lacks a closed-loop system architecture that deeply integrates robust visual perception, intelligent multi-arm collaboration, and hard real-time control. In real logistics scenarios, this results in significant limitations in the adaptability, stability, and execution certainty of existing solutions due to dynamic complexity, drastic environmental changes, and a disconnect between algorithm planning and underlying execution. Furthermore, in the domain of 3D perception for unstructured environments, Smith et al. [18] in their work “Geometry‐Aware 3D Point Cloud Learning for Precise Cutting‐Point Detection in Unstructured Field Environments” proposed an innovative solution for precise manipulation tasks in field environments using a geometry-aware point cloud learning technique. Their research demonstrates the effectiveness of integrating geometric priors with deep learning in complex, unstructured scenes. Although our current study primarily addresses dynamic grasping in structured logistics settings using RGB visual information, the work by Smith et al. provides a valuable reference and potential direction for future integration of multi-modal perception (e.g., RGB-D point clouds) to further enhance system robustness in highly cluttered logistics scenarios.

In summary, many scholars have researched logistics mechanical arms and vision algorithms, but there is currently no dynamic grasping method that collaborates between vision algorithms and mechanical arms. In view of this, the study proposes a dynamic grasping system that collaborates between a visual algorithm and a mechanical arm. This system reduces the error of mechanical arm grasping in logistics and improves grasping efficiency. The goal is to provide theoretical support for grasping in a logistics production line.

## 3. Methods

### 3.1 Design of dynamic gripping system for logistics production line

**Fig 1:**
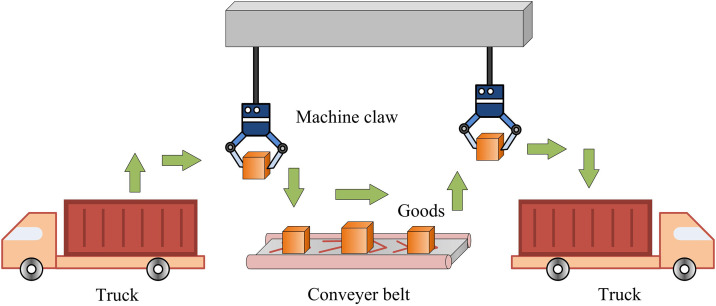
Logistics production line transportation process.

A logistics production line usually consists of several links, including material transportation, sorting, packaging and storage. In these segments, dynamic gripping systems play a crucial role in ensuring that materials can be efficiently and accurately transferred from one segment to another [19]. Dynamic gripping system is a complex and highly automated system, which involves the cooperative work of multiple links and equipment. In a logistics production line, all aspects of transportation, sorting, packaging and storage of items need to be carried out efficiently and accurately. The core objective of dynamic gripping and motion planning is to accurately grip boxes while ensuring obstacle avoidance and smoothly transport them to the designated locations [20,21]. The key process is shown in Fig 1.

In Fig 1, the operation of the dynamic gripping system involves real-time detection of the position of the box, path planning of the mechanical arm, and precise gripping control. During the transportation of an item, its spatial coordinates are first determined. Subsequently, the mechanical arm operates according to a preset path while taking into account the need for obstacle avoidance to ensure that the mechanical arm does not collide with other equipment or obstacles on the production line during its movement. When approaching the box, the mechanical arm fine-tunes the position and attitude of its end-effector to ensure that it can accurately and stably grasp the box. Finally, the mechanical arm transports the box smoothly to the designated place to complete the whole gripping task [22]. Interceptive capture uses the target container’s current orientation, rate, and acceleration to predict its future position. Then, it designs the robot’s movement to ensure it can intersect the target object. The linear uniform motion equation of the box is shown in Equation (1).


$$t_{cg}=(\begin{array}{c} {}*20cx_{c_{g}}-x_{c_{0}} \end{array})/V_{c}$$ (1)

In Equation (1), $t_{cg}$ indicates the planned capture moment. The $x_{c_{g}}$ represents the position of the target object on the x axis at the $x_{c_{0}}$ moment relative to the global coordinate system. denotes the initial position of the target box. $V_{c}$ denotes the uniform motion speed of the target box on the conveyor belt. The motion of the robot end is a nonlinear non-uniform motion equation as shown in Equation (2) [23].


$$S=\sqrt{{\left(x_{0}-x_{cg}\right)}^{2}+{\left(y_{0}-y_{cg}\right)}^{2}+{\left(z_{0}-z_{cg}\right)}^{2}}$$ (2)

In Equation (2), S denotes the end movement distance. $x_{0}$, $y_{0}$, and $z_{0}$ denote the initial position of x axis, y axis, and z axis, respectively. $y_{c_{g}}$ and $z_{c_{g}}$ denote the predicted positions of the target box in y and z axes, respectively. The expression for the segmented function of the time required for the axis robot to move from the initial position to the grasping point is shown in Equation (3).


$$t_{bg}=\left\{ \begin{array}{c} \sqrt{4S}/a_{b},S⩽V_{b}^{\text{2}}/2a_{b}\\ S/V_{b}+V_{b}/a_{b},S>V_{b}^{\text{2}}/2a_{b} \end{array}\right.$$ (3)

**Fig 2:**
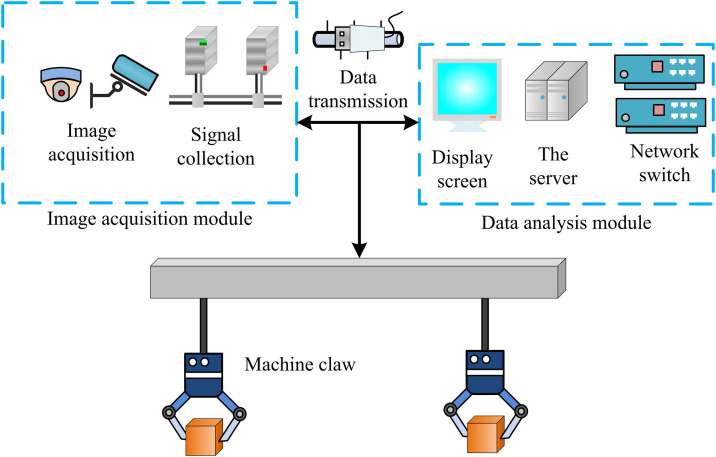
Dynamic capture system hardware and software composition.

In Equation (3), $t_{bg}$ denotes the total time required for the robot end to move from the initial position to the grasping point. $a_{b}$ denotes the maximum acceleration of the robot end. $V_{b}$ denotes the maximum velocity of the robot end. $V_{b}^{2}/2a_{b}$ denotes the minimum distance required for the robot to accelerate to $V_{b}$ with acceleration $a_{b}$. To realize the complete dynamic grasping, a complete hardware and software collaborative system architecture needs to be constructed. The hardware and software composition of the dynamic grasping system designed in the study is shown in Fig 2.

In Fig 2, the software and hardware of the dynamic grasping system designed in the study includes modules such as industrial camera, image acquisition card, mechanical arm, controller and end-effector. The industrial camera is responsible for capturing the image information of the target object, and the image acquisition card transmits the image information to the controller for processing. The controller analyzes the image information based on the vision algorithm, determines the position, attitude and other information of the target object, and plans the movement trajectory of the mechanical arm. The mechanical arm moves according to the instructions of the controller to realize the precise grasping of the target object [24,25]. The end-effector is designed according to the different shapes, sizes and other characteristics of the grasping objects to ensure the stability and accuracy of the grasping.

### 3.2 Improvement of dynamic grasping system for logistics production based on mechanical arm collaboration

**Fig 3:**
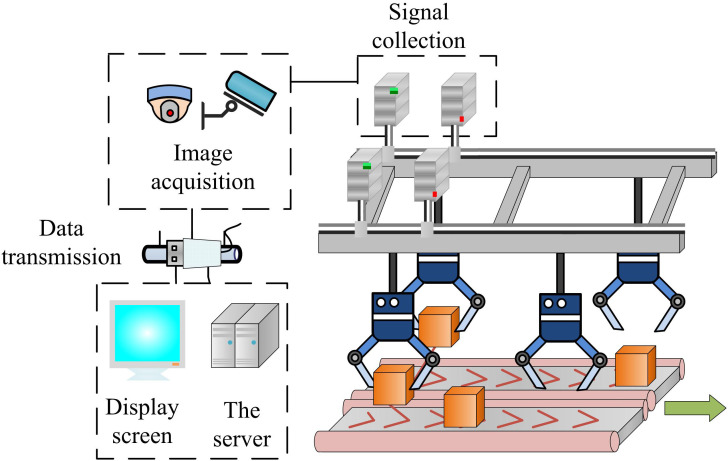
Operation process of dynamic grasping system based on robotic arm collaboration.

In complex or specialized scenarios, it is sometimes difficult for dynamic gripping systems to fully adapt to all the requirements of a logistics production line. The mechanical arm collaboration mechanism involves multiple mechanical arms working together on a logistics production line to perform complex gripping and handling tasks. This type of collaboration can effectively improve productivity and enhance the adaptability and flexibility of the system [26]. Therefore, to further optimize the flexibility and adaptability of the system, the mechanism of mechanical arm collaboration is introduced in the study. Through the close cooperation between the mechanical arm and the dynamic grasping system, a more efficient and accurate grasping operation of the target object is realized. The operation flow of the dynamic grasping system based on mechanical arm collaboration is shown in Fig 3.

In Fig 3, under the framework of mechanical arm collaboration, each mechanical arm is equipped with independent controllers and sensors to ensure that they can sense the surrounding environment in real time and make adjustments accordingly. Meanwhile, these mechanical arms share information and coordinate with each other through a communication network to realize the optimal assignment and execution of tasks. In the process of realizing mechanical arm collaboration, to precisely control the movement of the mechanical arm, the study first determines the movement axis and coordinate system of each joint. To achieve precise control of the robotic arm’s movements and lay the groundwork for collaboration, a kinematic model of a single robotic arm is first established using Denavit-Hartenberg (D-H) parameters, as shown in Equation (4).


$$T_{i}=\left[\begin{array}{cccc} {}*20c\cos\theta_{i} & -\sin\theta_{i}\cos\alpha_{i} & \sin\theta_{i}\sin\alpha_{i} & a_{i}\cos\theta_{i}\\ \sin\theta_{i} & \cos\theta_{i}\cos\alpha_{i} & -\cos\theta_{i}\sin\alpha_{i} & a_{i}\sin\theta_{i}\\ 0 & \sin a_{i} & \cos a_{i} & d_{i}\\ 0 & 0 & 0 & 1 \end{array}\right]$$ (4)

In Equation (4), $\theta_{i}$ denotes the joint angle. $\alpha_{i}$ denotes the joint length. $\alpha_{i}$ denotes the joint twist angle. $d_{i}$ denotes the joint offset. To realize the collaborative operation of multiple mechanical arm, the inverse kinematics problem needs to be solved. The collaborative mechanical arm inverse kinematics solution is shown in Equation (5).


$$\theta^{x}=argmin_{-}\theta [\alpha_{1}\cdot ||J(\theta )\cdot \Delta x-\Delta x_{d}|{|}^{2}+\alpha_{2}\cdot ||\theta_{n}|{|}^{2}+\alpha_{3}\cdot P_{coll}||\theta_{env}|{|}^{2}+\alpha_{4}\cdot ||\theta |{|}^{2}]$$ (5)

In Equation (5), $\theta^{x}$ denotes the optimal joint angle vector. J(θ) denotes the kinematic mapping. $\Delta x-\Delta x_{d}$ denotes the amount of change in end-execution target position. $\theta_{n}$ denotes the mechanical arm neutral position. $P_{coll}$ denotes collision penalty coefficient. $\theta_{env}$ denotes the set of environmental obstacles. θ denotes joint angular velocity vector. $\alpha_{1}$, $\alpha_{2}$, $\alpha_{3}$, and $\alpha_{4}$ denote optimization weight coefficients, respectively. In dynamic grasping scenarios, accurate prediction of target position is crucial. The dynamic target prediction grasping point calculation for mechanical arm collaboration is shown in Equation (6).


$$G_{target}(t)=P_{target}(t_{0})+V_{target}(t_{0})\cdot (t-t_{0})+0.5\cdot A_{target}(t_{0})\cdot (t-t_{0}{)}^{2}$$ (6)

In Equation (6), $G_{target}(t)$ denotes the position of the target grasping point at the t moment. $P_{target}(t_{0})$ denotes the initial position of the target at the $t_{0}$ moment. $V_{target}(t_{0})$ denotes the target velocity at $t_{0}$ moment. $A_{target}(t_{0})$ denotes the target acceleration at $t_{0}$ moment. t denotes the prediction time point. $t_{0}$ denotes the initial observation time. To ensure the stability of the grasping process, it is necessary to reasonably allocate the grasping force of the mechanical arm. The mechanical arm collaborative grasping force allocation is calculated as shown in Equation (7).


$$\begin{array}{c} \min{\mathrm{\backslash nolimits}}_{f}\Sigma_{i}(w_{i}\cdot ||f_{i}||),\left\{ \begin{array}{c} W\cdot f=F_{ext}+m\cdot g\\ G\cdot f\geq 0\\ f_{\min}\leq f_{i}\leq f_{\max} \end{array}\right. \end{array}$$ (7)

In Equation (7), f denotes the contact force vector. $w_{i}$ denotes the i th contact point weight. W denotes the force mapping matrix. $F_{ext}$ denotes the external disturbance force. m·g denotes the object gravity. G denotes the friction constraint. $f_{\min}$ and $f_{\max}$ denote minimum and maximum output forces, respectively. Among them, $W\cdot f=F_{ext}+m\cdot g$ denotes the force balance constraint. G·f≥0 denotes friction force constraint. $f_{\min}\leq f_{i}\leq f_{\max}$ denotes output force constraint. To realize the efficient collaboration of multi-mechanical arm, a scientific task allocation mechanism is required. The dynamic task allocation expression for mechanical arm collaboration is shown in Equation (8).


$$A_{\min}=\;[T_{k}+\sum {\mathrm{\backslash nolimits}}_{j}\left(||p_{k}-p_{j}||v_{k})\right],\left\{ \begin{array}{c} \sum {\mathrm{\backslash nolimits}}_{k}A_{jk}=1\\ A_{jk}\in \{0,1\} \end{array}\right.$$ (8)

In Equation (8), $T_{k}$ denotes the current task queue time of the mechanical arm. $A_{jk}$ denotes the task assignment matrix. $p_{k}$ denotes the current position of the mechanical arm. $p_{j}$ denotes the mechanical arm target position. $v_{k}$ denotes mechanical arm average moving speed. This task allocation model aims to minimize the total completion time for all robotic arms while considering their current positions and mobility. This achieves efficient collaborative scheduling. In the dynamic grasping process, real-time attitude correction is essential for ensuring grasping accuracy. Equation (9) shows the real-time posture correction calculation during collaborative grasping with a robotic arm.


$$\left\{ \begin{array}{c} \Delta G=K_{p}\cdot e(t)+K_{d}\cdot de(t)/dt\\ e(t)=G_{obs}(t)-G_{pred}(t) \end{array}\right.$$ (9)

In Equation (9), ΔG denotes the amount of grab point position correction. e(t) denotes the grasping point prediction error. $G_{obs}(t)$ denotes the real-time observation of the grasping position by the vision system. $G_{pred}(t)$ denotes the motion model predicted grasping point position. $K_{p}$ denotes scale factor. $K_{d}$ denotes differential gain coefficient. A comprehensive energy efficiency evaluation system is required to evaluate the performance of the system. The energy efficiency evaluation calculation for collaborative grasping by mechanical arm is shown in Equation (10) [27].


$$\eta =[\lambda_{succ}\cdot N_{total}]/[T_{total}\cdot (E_{total}+P_{coll}\cdot C_{coll})]$$ (10)

**Fig 4:**
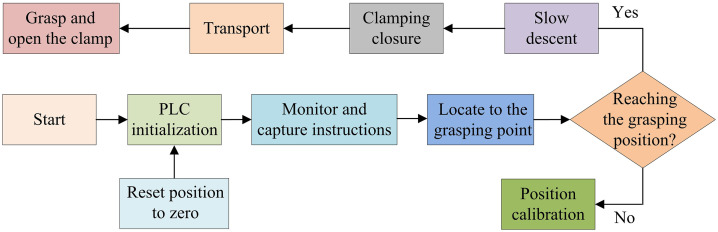
Operation process of automatic control of grasping motion of industrial robot arm based on PLC.

In Equation (10), η denotes the combined energy efficiency of the system. $\lambda_{succ}$ denotes the success rate of crawling. $N_{total}$ denotes the total number of crawling tasks. $T_{total}$ denotes the total task completion time. $E_{total}$ denotes total system energy consumption. $C_{coll}$ denotes the number of mechanical arm collisions. To further improve the automation performance of the dynamic gripping system for logistics production based on mechanical arm collaboration, the study uses PLC to automate the dynamic gripping system. The system generates high-precision joint motion parameters by receiving dynamic target position, grasp timing, and collaboration commands from the multi-robot system in real time and combining them with the preset collaborative decision-making algorithm [28,29]. Based on these parameters, the system can calculate the angles and displacements that should be achieved by each joint and convert them into motor control commands. The PLC-based automated control operation flow of mechanical arm dynamic gripping motion is shown in Fig 4.

In Fig 4, the system starts with the initialization procedure of the PLC while driving the wrist disc spring mechanism to reset to zero automatically. Subsequently, the PLC continuously monitors the gripping command signal. Once the command signal is detected, the mechanical arm moves to a safe position above the gripping point and descends at a slow rate. When the preset gripping height is reached, the gripper jaws will perform a closing action. Once gripping is complete, the workpiece will be transported to the target position and the mechanical arm will release the gripper jaws after a slow descent to complete placement. Finally, the system resets and waits for the next command. The PLC controls the timing of the opening and closing of the gripper jaws and the movement of the mechanical arm. Through this process, the PLC achieves highly efficient and precise automation control of the gripping movement of industrial robot arms.

### 3.3 Optimization of mechanical arm dynamic gripping system based on improved vision algorithm

**Fig 5:**
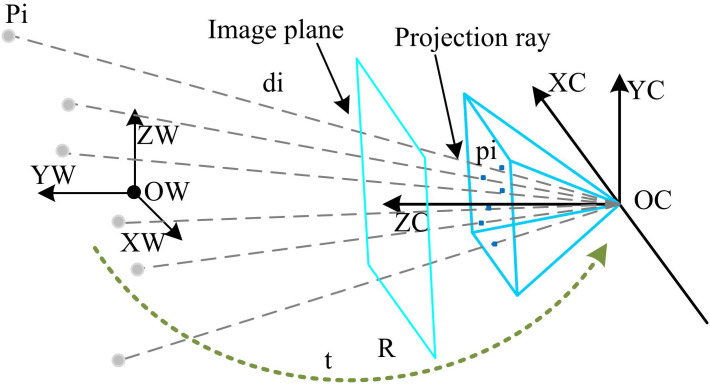
Schematic diagram of PnP principle.

The dynamic gripping system for logistics production based on mechanical arm collaboration has achieved certain automation results in practical applications, but still faces many limitations. The system’s ability to adapt to complex environments and changing working conditions must be improved. Light changes, object blockages, and irregular shapes often affect the gripping accuracy and stability of the mechanical arm. Therefore, the study introduces an improved PnP vision algorithm to optimize the dynamic grasping system. The algorithm significantly improves the target localization accuracy by enhancing the feature robustness and introducing a dynamic error compensation mechanism. Through finer image processing and analysis, the recognition and localization accuracy of the target object is improved, which makes the mechanical arm more flexible and efficient in the dynamic grasping process [30,31]. The schematic diagram of PnP principle is shown in Fig 5.

The basic principle of the PnP original algorithm, shown in Fig 5, contains two key reference systems, the world coordinate system (origin Ow, axes Xw and Zw) and the camera coordinate system (optical axis Zc). The 3D spatial point Pi in the Fig 5 is defined in the world coordinate system, and the transformation from the world coordinate system to the camera coordinate system is realized by the rotation matrix R (shown by the arrow in the Fig 5). The mathematical relationship is [Xc;Yc;Zc]=R-[Xw;Yw;Zw]+t, where t is the translation vector. The core of the PnP algorithm involves solving for the camera’s position relative to the world coordinate system by establishing a correspondence between known 3D spatial points and their 2D projections in the image. This forms the theoretical basis for achieving precise positioning of the mechanical arm’s vision system. Its algorithmic feature weighting defines the feature point confidence, as shown in Equation (11).


$$w_{i}=\alpha \cdot S_{SIFT}(i)+\beta \cdot C_{edge}(i)+\gamma \cdot I_{inv}(i)$$ (11)

In Equation (11), $w_{i}$ denotes the combined weight value. α, β, and γ denote the weight coefficients. $S_{SIFT}(i)$ denotes scale invariant feature transform. $C_{edge}(i)$ denotes edge detection gradient value. $I_{inv}$ denotes intensity invariant. The feature points are grouped by spatial distribution to solve the calculation, as shown in Equation (12).


$$P_{opt}=\sum_{k=1}^{K}w_{k}\cdot P_{k},\;w_{k}=\frac{inliers_{k}}{\sum inliers}$$ (12)

In Equation (12), $P_{opt}$ denotes the optimal model parameters. K denotes the number of sampling iterations. $w_{k}$ denotes the weight corresponding to the k th group of model assumptions $P_{k}$. $P_{k}$ denotes the model parameter assumptions. $inliers_{k}$ denotes the number of interior points. Next, the motion blur error due to conveyor belt vibration is suppressed. Its visual-motion coupling correction is calculated as shown in Equation (13).


$$\Delta p=k_{p}\cdot e_{v}+k_{d}\cdot \frac{de_{v}}{dt}$$ (13)

In Equation (13), Δp denotes the increment of the control quantity. $k_{p}$ denotes the proportionality coefficient. $e_{v}$ the error of the current system. $k_{d}$ the differential coefficient denotes. $\frac{de_{v}}{dt}$ denotes the differentiation of the error. The initial values of these parameters were estimated theoretically based on the desired response characteristics of the system to a step input. They were subsequently fine-tuned on both simulation and physical platforms using the Ziegler-Nichols method to achieve a balance between response speed and overshoot. Then, the grab point selection strategy with the introduction of energy efficiency constraints is calculated as shown in Equation (14).


$$p_{grasp}^{*}=\underset{p}{\mathrm{\backslash arg}\min}\left(\lambda_{1}||p-p_{opt}|{|}^{2}+\lambda_{2}\cdot \eta (p)\right)$$ (14)

In Equation (14), $p_{grasp}^{*}$ denotes the optimal grasping parameters. $\underset{p}{\mathrm{\backslash arg}\min}$ denotes the variable p that is found to minimize the latter function. $\lambda_{1}$ and $\lambda_{2}$ denote the weighting coefficients. $||p-p_{opt}|{|}^{2}$ denotes the paradigm of positional deviation. η(p) denotes the grasping cost function. Finally, feature pre-screening is used to accelerate the computation as shown in Equation (15).


$$t_{proc}\leq \frac{t_{pred}-t_{comm}}{N_{feat}}$$ (15)

**Fig 6:**
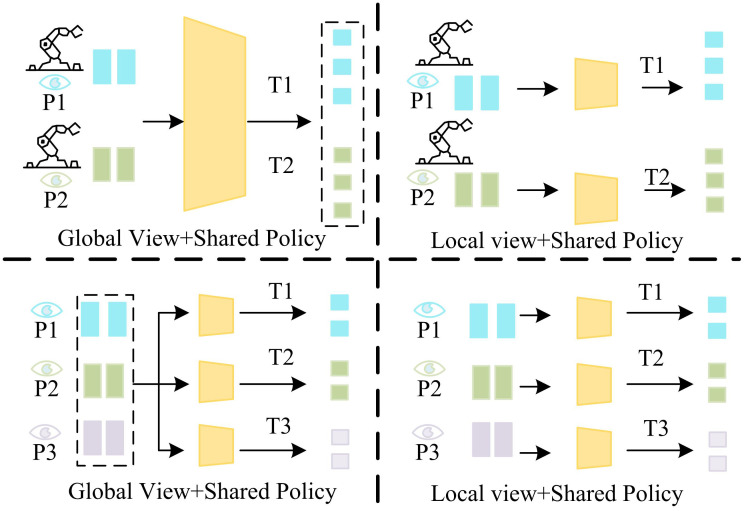
Dynamic capture with MFW-PnP fusion.

In Equation (15), $t_{proc}$ denotes the task processing time. $t_{pred}$ denotes task prediction completion time. $t_{comm}$ denotes communication delay time. $N_{feat}$ denotes the number of parallel feature tasks. In the study of cooperative control strategies for dynamic grasping systems, the optimal design of the multi-view task allocation mechanism is the key link to achieve efficient operations. By integrating global-local synergy with individual execution efficiency, the research has developed intelligent decision-making systems that are adapted to the dynamic environment. The core of the system is to balance the relationship between overall task allocation and local path planning, and to ensure the collaborative operation capability of multi-mechanical arm systems under complex working conditions through hierarchical optimization strategies. Its dynamic grasping system cooperative work based on multi-feature weighted PnP algorithm is shown in Fig 6.

**Fig 7:**
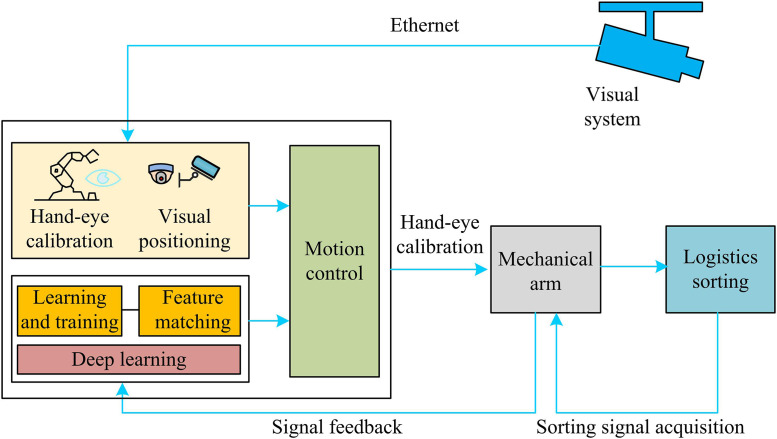
General system flow chart.

Fig 6 shows how the dynamic grasping system, which is based on a multi-feature weighted PnP algorithm, works together. A hierarchical structure is used to compare the task allocation mechanism from global and local perspectives. From a global perspective, the system coordinates the distributions of task nodes (T1-T3) and grasping points (P1-P2) and schedules the cooperative work of multiple mechanical arms. While in the local perspective, it focuses on the path planning of individual mechanical arm (e.g., the execution sequence of T1 → P1 → P2) to optimize the local motion trajectory while maintaining the strategy sharing. The study constructs a complete dynamic grasping system integration system, which realizes the closed-loop process from visual perception to mechanical arm control through multi-level synergy. Its flowchart is shown in Fig 7.

In Fig 7, the system adopts an innovative modular architecture to realize intelligent logistics sorting through the organic synergy of four functional units. The visual localization unit builds an accurate coordinate conversion system with the help of hand-eye calibration technology, and completes the real-time identification and localization of target objects with the combination of deep learning algorithms. Intelligent decision-making unit relies on learning and training mechanism and forward matching module to dynamically optimize the sorting strategy and mechanical arm cooperative path planning. The motion control unit accurately drives the mechanical arm to execute the sorting operation according to the visual localization results. Meanwhile, the signal feedback loop continuously collects operation data to realize the whole process monitoring. The system builds a complete closed-loop workflow from visual perception, intelligent decision-making to motion execution and status feedback. Each functional module maintains real-time data interaction through high-speed Ethernet to ensure high precision and efficiency of the sorting operation.

The main control loop of VRCDS system pseudocode is shown in Table 1.

**Table 1:** The main control loop of VRCDS system pseudocode.

Algorithm 1: VRCDS Main Control Loop
Input: Camera stream, Conveyor speed v_conveyor Output: System performance metrics 1: // Initialize system components 2: vision_system InitializeVision() 3: robot_arms InitializeRobots() 4: plc_controller ← InitializePLC() 5: 6: while system_running do 7: // Perception phase 8: image ← CaptureImage() 9: objects ← DetectObjects(image) 10: poses ← EstimatePoses(objects)// Using the multi-feature weighted PnP algorithm from Section 3.3 11: 12: // Prediction & planning phase 13: for each object in objects do 14: grasp_point ← PredictGraspPoint(object, v_conveyor)// Using the dynamic target prediction model from Eq. (6) 15: tasks.Add(grasp_point) 16: end for 17: 18: // Multi-arm coordination 19: assignment ← AllocateTasks(tasks, robot_arms)// Using the dynamic task allocation optimizer from Eq. (8) 20: 21: // Execution phase 22: for each robot in robot_arms do 23: assigned_tasks ← GetAssignedTasks(assignment, robot) 24: for each task in assigned_tasks do 25: success ← ExecuteGrasp(robot, task, plc_controller)// Invoking the PLC control flow (Fig 4) and IK solver (Eq. (5)) from Section 3.2 26: LogPerformance(success) 27: end for 28: end for 29: 30: // Energy efficiency monitoring (Eq. 10) 31: efficiency ← CalculateEnergyEfficiency() 32: UpdateMetrics(efficiency) 33: end while

## 4. Results

### 4.1 Performance testing of a dynamic grasping system based on collaboration between vision algorithms and mechanical arm

To validate the performance of the research designed dynamic grasping system based on the collaboration of vision algorithm and mechanical arm. The study refers to the model as VRCDS. the study uses Dynamic Grasping Benchmark Dataset (DGBD) and YCB-Video Dynamic Subset (YDS) for testing. Among them, the DGBD dataset (https://berkeleyautomation.github.io/dex-net/) contains a variety of sequences of common objects moving on conveyor belts at different speeds. The YDS (YCB-Video Dataset) dataset (https://rse-lab.cs.washington.edu/projects/posecnn/) is based on the well-known YCB-Video object dataset, which filters out objects suitable for grasping. By placing an object on a speed adjustable conveyor belt, video sequences of it in motion are recorded and its positional changes are accurately labeled. The basic hardware and software environment setup for the experiments used in the study is shown in Table 2.

**Table 2:** The experimental basic environmental parameters.

Type	Component	Specification	Key feature
Hardware	Robotic arm	UR5e (5 kg, ± 0.03 mm)	High-precision motion
	Gripper	Robotiq 2F-140	Adaptive grasping
	Camera	Basler acA2440-75um (75fps)	High-speed RGB capture
	Computer	i9-13900K + RTX 4090	Real-time AI processing
	Conveyor	0.1-1.5 m/s adjustable	Dynamic object movement
Software	OS	Ubuntu 20.04 + ROS 2 Humble	Robotics middleware
	Vision	OpenCV 4.8 + PyTorch 2.0	Object detection/grasping
	Control	MoveIt 2	Motion planning
	Calibration	EasyHandeye	Camera-robot alignment

**Fig 8:**
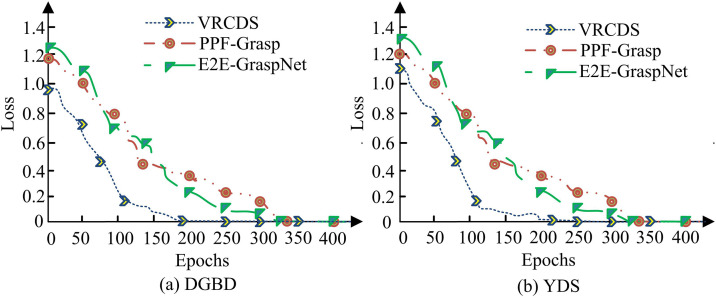
Training loss curves on the DGBD and YDS datasets.

Table 2 contains the computer hardware configurations used for the experiments, including key parameters such as processor model, memory size, and storage device type. The study analyzes the training loss of VRCDS with PPF-based pose estimation & grasp planning (PPF-Grasp) and end-to-end 6-DoF grasp generation network (E2E-GraspNet) in the dataset, as shown in Fig 8.

**Fig 9:**
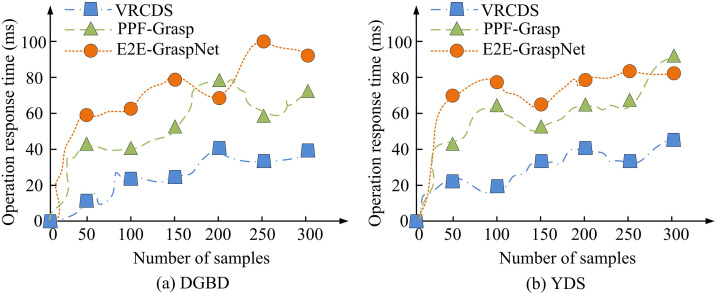
Different methods of encryption response speed.

In Fig 8(a), on the DGBD dataset, the initial loss value of the VRCDS method is about 0.9, which gradually converges to 0 and remains stable after 200 iterations. The PPF-Grasp method has an initial loss of about 1.19, which converges to 0 and remains stable after 330 iterations. The E2E-GraspNet method has an initial loss of about 1.26, which converges to 0 and remains stable after 340 iterations. In Fig 8(b), on the YDS dataset, the initial loss value of the VRCDS method is about 1.12, which converges to 0 and remains stable after 210 iterations. The PPF-Grasp method has an initial loss of about 1.22, which converges to 0 and remains stable after 115 iterations. The E2E-GraspNet method has an initial loss of about 1.32, which converges to 0 and remains stable after 320 iterations. The results show that the loss values of the VRCDS method are significantly lower than those of the PPF-Grasp and E2E-GraspNet methods for the same number of training iterations and for both datasets. This demonstrates the VRCDS method’s superiority in terms of training effectiveness and convergence speed. The study analyzes the running response speed of different methods as shown in Fig 9.

**Fig 10:**
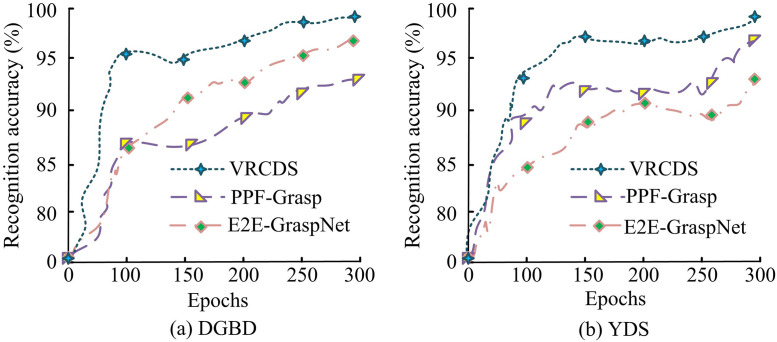
Recognition accuracy of different methods.

In Fig 9(a), on the DGBD dataset, as the number of samples increases, the response time of each method grows accordingly. When the number of samples reaches 300, the response time of the VRCDS method is about 39 ms, the PPF-Grasp method is about 71 ms, and the E2E-GraspNet method is about 87 ms. In Fig 9(b), an increase in the number of samples leads to a synchronous increase in the response times of various methods. On the YDS dataset, when the number of samples is 300, the response time of the VRCDS method is approximately 42 ms, that of the PPF-Grasp method is approximately 84 ms, and that of the E2E-GraspNet method is approximately 81 ms. In summary, with the same sample size, the VRCDS method has a significantly lower runtime response time than the PPF-Grasp and E2E-GraspNet methods, demonstrating faster response efficiency. The study compares the recognition accuracy of different methods, as shown in Fig 10.

In Fig 10, as the number of iterations increases, the recognition accuracy of different methods gradually improves. In Fig 10(a), on the DGBD dataset, the VRCDS method achieves a recognition accuracy of up to 97.5% after 300 iterations. Next is the E2E-GraspNet method. At the same number of iterations, its recognition accuracy is 94.4%. The PPF-Grasp method achieves a recognition accuracy of 92.1% after 300 iterations. In Fig 10(b), on the VRCDS method achieved an object detection accuracy of 98.2% on the YDS dataset. This metric reflects the system’s ability to reliably locate and identify target objects. It should be noted that successful dynamic grasping relies not only on detection but also on precise 6D pose estimation, the latter being the core contribution of our multi-feature weighted PnP algorithm. The PPF-Grasp method follows closely behind with an identification accuracy of 95.9%. The E2E-GraspNet method achieves an identification accuracy of 92.4% after 250 iterations. Overall, the VRCDS method proposed in this study demonstrates superior performance in terms of identification accuracy.

### 4.2 Application analysis of a dynamic gripping system based on visual algorithms and mechanical arm collaboration

**Fig 11:**
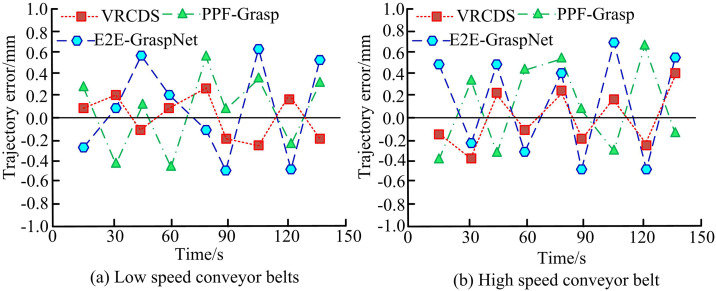
Error curve of mechanical arm under conveyor belt at different speeds.

To validate the applicability of the model in real-world scenarios, the study selects a logistics sorting centre to conduct an application analysis of dynamic parcel sorting. The study selects parcels of various shapes, sizes, and weights and analyses the actual application performance of the system under low and high speed conveyor belts. The mechanical arm grasping error curves under different speed conveyor belts are shown in Fig 11.

**Fig 12:**
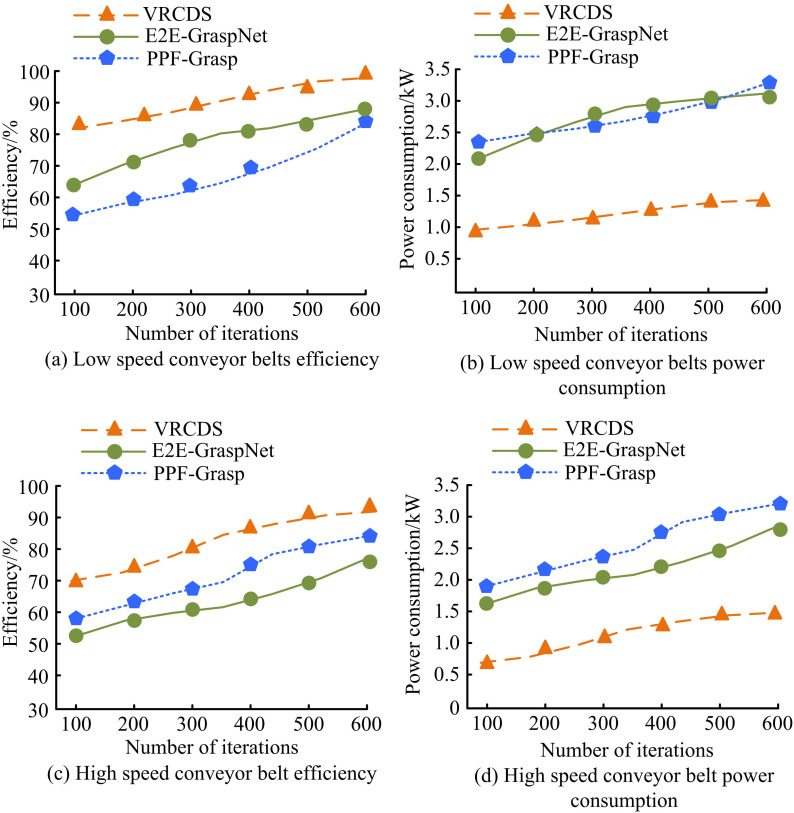
Power consumption, grasping efficiency and error of mechanical arm grasping.

In Fig 11(a), under low-speed conveyor belt conditions, the trajectory error of VRCDS fluctuates between −0.2 mm and 0.2 mm, with relatively small fluctuations and a relatively smooth curve. The fluctuation range of PPF-Grasp is approximately −0.5 mm to 0.6 mm, with a larger fluctuation amplitude. The error of E2E-GraspNet is roughly between −0.5 mm and 0.8 mm, with a large fluctuation amplitude and a highly fluctuating curve. In Fig 11(b), the trajectory error of VRCDS fluctuates between −0.4 mm and 0.4 mm, with small fluctuations and a relatively smooth curve. The fluctuation range of PPF-Grasp is approximately −0.7 mm to 0.4 mm, with larger fluctuations. The error of E2E-GraspNet is roughly between −0.6 mm and 0.8 mm, with large fluctuations and a highly fluctuating curve. Overall, VRCDS maintains good trajectory accuracy under both conveyor belt speeds, outperforming the E2E-GraspNet and PPF-Grasp methods. The study compares the power consumption, grasping efficiency, and error of the mechanical arm grasping under different conveyor belt speeds after continuous operation for 1 hour, as shown in Fig 12.

**Fig 13:**
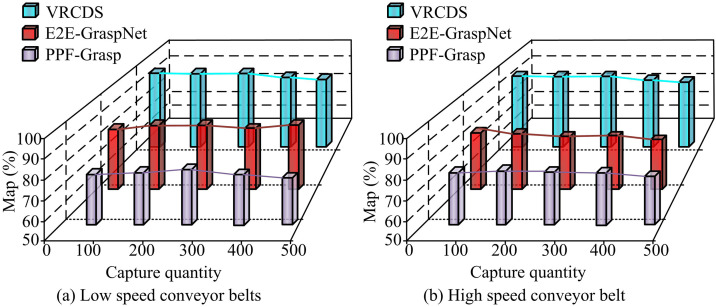
Mean average precision of different algorithms.

In Fig 12(a), under low-speed conditions, the efficiency of VRCDS remains the highest as the number of iterations increases, reaching approximately 98% at 600 iterations. E2E-GraspNet follows, reaching approximately 82% at 600 iterations. PPF-Grasp has the lowest efficiency, reaching approximately 80% at 600 iterations. In Fig 12(b), under low-speed conditions, PPF-Grasp has the highest power consumption, reaching approximately 3.3 kW at 600 iterations. E2E-GraspNet is at an intermediate level, reaching approximately 3.0 kW at 600 iterations. VRCDS has the lowest power consumption, reaching approximately 1.3 kW at 600 iterations. In Fig 12(c), under high-speed conditions, VRCDS still achieves the highest efficiency, approximately 96% at 600 iterations. E2E-GraspNet has a relatively lower efficiency, approximately 72% at 600 iterations. PPF-Grasp has the lowest efficiency, approximately 82% at 600 iterations. In Fig 12(d), under high-speed conditions, PPF-Grasp has the highest power consumption, approximately 3.2 kW at 600 iterations. E2E-GraspNet is at an intermediate level, approximately 2.7 kW at 600 iterations. VRCDS has the lowest power consumption, approximately 1.3 kW at 600 iterations. The results show that the VRCDS method is highly efficient and has low power consumption under both low- and high-speed conveyor belt conditions. Its performance advantages become more pronounced as the number of iterations increases. The study compares the mean average precision (mAP) of different methods, as shown in Fig 13.

**Fig 14:**
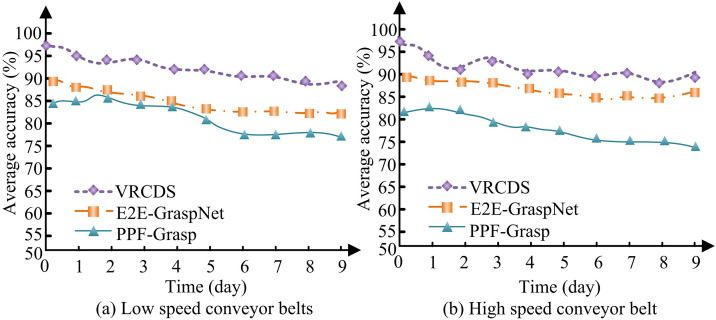
Capture the average accuracy.

In Fig 13(a), under low speed conveyor belt conditions, the Map of VRCDS always remains above 90%. The Map of E2E-GraspNet fluctuates between 80% and 90%. The Map of PPF-Grasp is the lowest, only between 70% and 80%. In Fig 13(b), under high-speed conveyor belt conditions, the VRCDS map remains at around 90%. The E2E-GraspNet map fluctuates at around 80%. The PPF-Grasp map is the lowest, fluctuating at only around 70%. The results proves that the research methods have strong generalization ability. The study compares the average accuracy of crawling for different methods running continuously for 9 days as shown in Fig 14.

In Fig 14(a), the average accuracy of grasping for VRCDS under low speed conveyor belt conditions decreases from an initial 97.82% to 91.57% after 9 days. The average accuracy of E2E-GraspNe’s grasping decreases from 90.07% initially to 80.37% after 9 days. The average accuracy of PPF-Grasp’s grabs decreases from an initial 84.83% to 77.69% after 9 days. In Fig 14(b), the average accuracy of grasping for VRCDS under high speed conveyor belts condition decreased from initial 96.91% to 90.58% after 9 days. The average accuracy of E2E-GraspNe’s grasping decreases from an initial 89.84% to 85.37% after 9 days. The average accuracy of PPF-Grasp’s grasping decreases from an initial 82.06% to 74.79% after 9 days. The results show that VRCDS runs more stably and has a higher average accuracy of crawling. To evaluate the adaptability and robustness of the VRCDS system under non-ideal working conditions, the study, based on benchmark tests, simulates three common challenging scenarios in logistics sorting centers: (1) Local occlusion, simulating improper stacking or placement of items. (2) Light variation, simulating uneven lighting caused by weather or indoor lighting. (3) Interference from similar objects, simulating similar items existing in complex sorting tasks. The system performs at least 50 crawling tasks in each scenario. The statistical results of its core performance indicators (crawling success rate, trajectory error, and time consumption per crawling cycle) are shown in Table 3.

**Table 3:** Performance of VRCDS system under challenging scenarios.

Test Scenario	Grasping success rate (%)	Mean trajectory error (mm)	Mean cycle time (s)
Baseline	98.0	0.5	2.1
A: Partial occlusion	91.5	0.8	2.4
B: Lighting variation	88.0	0.10	2.5
C: Similar object interference	85.0	0.7	2.7

As shown in Table 3, the VRCDS system is fundamentally robust in various challenging scenarios. However, its performance is slightly lower than under benchmark conditions. In local occlusion scenarios, the system maintains a capture success rate of 91.5%, with only slight errors and increased time consumption. The influence of the lighting variation scene is the most significant. Due to the impaired feature stability, the success rate drops to 88.0% and the trajectory error is the largest. The system maintained good trajectory control capabilities in scenarios with interference from similar objects. However, the complex decision-making process resulted in a significant increase in time consumption and a decrease in the success rate to 85.0%. These results verifies the system’s basic functionality under non-ideal conditions and clarifies its limitations in extreme lighting and highly similar environments. This information can guide subsequent optimization efforts.

## 5. Discussion

The research results demonstrated that the VRCDS system proposed in this study significantly enhanced the performance of dynamic logistics grasping tasks overall. This improvement was achieved through a multi-feature weighted PnP vision algorithm, a multi-robotic arm collaborative control strategy, and PLC automation integration. This innovative system integrated visual perception, intelligent decision-making, and collaborative execution modules to form a closed-loop architecture with complementary advantages. The improved PnP algorithm achieved high-precision target positioning in complex environments by enhancing feature robustness and dynamic error compensation. The hierarchical collaborative control strategy optimized the tasks and paths of multiple robotic arms, enhancing the system’s efficiency and flexibility. The introduction of PLC ensured the stable and reliable automated operation of the entire process. Experiments revealed that this system outperformed other methods in important areas such as grasping accuracy, control of trajectory error, and energy consumption efficiency. It provided a practical, efficient, and precise technical solution for the dynamic grasping problem in logistics automation.

The VRCDS system has three obvious advantages over existing research. First, compared to traditional single-robotic-arm grasping systems, the multi-robotic-arm collaborative mechanism of VRCDS overcome the efficiency limitations of single-point operations. The reason lies in its hierarchical task allocation strategy, which enables the parallel processing of multiple objects. This significantly increases the overall system throughput, a capability lacking in single-arm systems. Convergence Speed: As quantitatively shown in Fig 8, our VRCDS method converged after approximately 200 and 210 iterations on the DGBD and YDS datasets, respectively. In contrast, the E2E-GraspNet method—representative of the end-to-learning paradigms discussed in [12,13]—required over 300 iterations to converge on the same datasets. This demonstrates a significant advantage in convergence speed.Data Efficiency: Data efficiency: Method [13] relies on a large amount of unlabeled data for pre-training (with high data requirements). In contrast, the multi-feature weighted PnP algorithm studied is based on geometric priors and completely eliminates this large-scale pre-training stage. Using only a regular-scale labeled training set (for example, the standard training split of the YDS dataset), the study method achieved a recognition accuracy rate of 98.2% (Fig 10). This combination of “less data dependence” and “higher performance output” clearly demonstrates the superiority of our method in data efficiency. This is achieved through feature confidence weighting and visual-motion coupling. Thus, the proposed method is more applicable in real industrial environments. Finally, differing from existing collaborative grasping frameworks that often focuses on algorithmic coordination, such as the holonic palletizing model by Borangiu et al. [11], this study deeply integrates a PLC-based hard real-time control layer. The key distinction is that the proposed system ensures the deterministic and stable execution of collaborative decision-making commands. This integration addresses the gap between high-level planning and low-level actuation, which is not fully covered in reference [11]. Its performance advantages, particularly with regard to trajectory accuracy and energy consumption control, are thoroughly verified through detailed experiments conducted in real-life logistics scenarios.

## 6. Conclusion

The VRCDS based on the collaboration of visual algorithms and robotic arms was studied, designed, and verified. This system successfully integrated a complete framework that incorporated a multi-feature weighted PnP vision algorithm, a multi-robotic arm hierarchical collaborative control strategy, and PLC automation. The experimental results showed that the system achieved a target recognition accuracy rate of 98.2%, controlled the trajectory error to within ±0.4 mm during the dynamic grasping process, and significantly reduced system power consumption. This research addressed the issues of insufficient grasping accuracy, the low collaborative efficiency of multiple arms, and the high energy consumption of existing technologies in complex, dynamic environments. The research provided an efficient, precise, and stable technical solution for upgrading logistics production lines intelligently.

## 7. Limitations and future work

There are several limitations in this study that need to be pointed out. First, the experimental verification is mainly carried out in a relatively controlled logistics sorting center environment. Therefore, the system’s stability and performance in extremely complex scenarios have not been fully verified. Such scenarios include the presence of dynamic obstacle interference, the high-speed movement of target objects, and reflection on the surface of special materials. Second, the current hardware configuration, which includes multiple industrial robot arms and high-end computing units, is relatively expensive. This cost factor may hinder the promotion of the system for small- and medium-sized industrial applications. Finally, this study primarily focuses on grasping regular, box-shaped items. Its ability to generalize to non-standard objects of various shapes and textures has not yet been verified.

Based on the current research results and identified limitations, future work will focus on the following directions: First, the new perception algorithms will be developed to effectively handle interference from dynamic obstacles, thereby enhancing the system’s adaptability in complex, dynamic environments. Secondly, in scenarios involving high-speed target objects, the dynamic target tracking and prediction algorithms will be optimized to ensure grasping accuracy and stability under these conditions. Finally, for the sake of expanding the application scope of the system, the visual feature extraction and grasping strategies for special material surfaces will be examined, and dedicated processing methods will be developed for highly reflective, transparent, and flexible objects. Meanwhile, there will be continued optimization of the system’s hardware architecture and control algorithms. This will reduce manufacturing costs while maintaining high performance, thereby enhancing the system’s applicability in a wider range of industrial scenarios.

## Supporting information

S1 Data Minimal data set definition. (DOCX)
