# Supplementary material for: Dynamic grasping system based on visual algorithm and robot arm collaboration in logistics production line
Source: PLoS One. 2026 Jan 9;21(1):e0340455. doi: 10.1371/journal.pone.0340455 (PMC12788689; doi:10.1371/journal.pone.0340455)
Supplement: S1 Data — (DOCX) [file pone.0340455.s001.docx]

**The data in Figure 8**

| Sample Size | Loss function on the DGBD dataset | | |
| --- | --- | --- | --- |
|  | VRCDS | PPF-Grasp | E2E-GraspNet |
| 0 | 0.9 | 1.19 | 1.26 |
| 50 | 0.75 | 1.01 | 1.12 |
| 100 | 0.53 | 0.82 | 0.75 |
| 150 | 0.15 | 0.45 | 0.61 |
| 200 | 0.05 | 0.38 | 0.25 |
| 250 | 0.04 | 0.23 | 0.18 |
| 300 | 0.03 | 0.18 | 0.10 |
| 350 | 0.02 | 0.02 | 0.02 |
| 400 | 0.01 | 0.01 | 0.01 |
| Sample Size | Loss function on the YDS dataset | | |
|  | VRCDS | PPF-Grasp | E2E-GraspNet |
| 0 | 1.12 | 1.22 | 1.32 |
| 50 | 0.78 | 1.01 | 1.15 |
| 100 | 0.53 | 0.82 | 0.75 |
| 150 | 0.18 | 0.47 | 0.61 |
| 200 | 0.05 | 0.38 | 0.25 |
| 250 | 0.04 | 0.23 | 0.18 |
| 300 | 0.03 | 0.18 | 0.10 |
| 350 | 0.02 | 0.02 | 0.02 |
| 400 | 0.01 | 0.01 | 0.01 |

**The data in Figure 9**

| Number of samples | Response speed of DGBD data set | | |
| --- | --- | --- | --- |
|  | VRCDS | PPF-Grasp | E2E-GraspNet |
| 0 | 0 | 0 | 0 |
| 50 | 10 | 40 | 59 |
| 100 | 20 | 39 | 60 |
| 150 | 20 | 50 | 78 |
| 200 | 38 | 75 | 65 |
| 250 | 35 | 55 | 95 |
| 300 | 39 | 71 | 87 |
| Epoch | Response speed of YDS data set | | |
|  | VRCDS | PPF-Grasp | E2E-GraspNet |
| 0 | 0 | 0 | 0 |
| 50 | 20 | 40 | 70 |
| 100 | 19 | 60 | 79 |
| 150 | 30 | 50 | 60 |
| 200 | 40 | 61 | 79 |
| 250 | 30 | 62 | 80 |
| 300 | 42 | 84 | 81 |

**The data in Figure 10**

| Epochs | DGBD data set recognition accuracy | | |
| --- | --- | --- | --- |
|  | VRCDS | PPF-Grasp | E2E-GraspNet |
| 0 | 0 | 0 | 0 |
| 100 | 95.1 | 85 | 88.5 |
| 150 | 94.8 | 87.5 | 88.6 |
| 200 | 96.1 | 90.1 | 91.5 |
| 250 | 97.1 | 91.1 | 93.5 |
| 300 | 97.5 | 92.1 | 94.4 |
| Epoch | YDS data set recognition accuracy | | |
|  | VRCDS | PPF-Grasp | E2E-GraspNet |
| 0 | 0 | 0 | 0 |
| 100 | 93.2 | 88.3 | 85.1 |
| 150 | 97.1 | 92.2 | 87.6 |
| 200 | 96.6 | 91.5 | 90.3 |
| 250 | 96.5 | 92.6 | 89.5 |
| 300 | 98.2 | 95.9 | 92.4 |

**The data in Figure 11**

| Time/s | Low speed conveyor condition grasping error curve | | |
| --- | --- | --- | --- |
|  | VRCDS | PPF-Grasp | E2E-GraspNet |
| 0 | 0 | 0 | 0 |
| 30 | 0.18 | -0.45 | 0.08 |
| 60 | 0.08 | -0.50 | 0.20 |
| 90 | -0.20 | 0.05 | -0.6 |
| 120 | 0.15 | -0.30 | -0.6 |
| 150 | -0.20 | 0.30 | 0.5 |
| Time/s | High speed conveyor belt condition grasping error curve | | |
|  | VRCDS | PPF-Grasp | E2E-GraspNet |
| 0 | 0 | 0 | 0 |
| 30 | -0.40 | 0.38 | -0.25 |
| 60 | -0.18 | 0.40 | -0.38 |
| 90 | -0.20 | 0.08 | -0.58 |
| 120 | -0.4 | 0.60 | -0.60 |
| 150 | 0.40 | -0.20 | 0.58 |

**The data in Figure 12**

| Number of samples | Low speed pickup efficiency | | |
| --- | --- | --- | --- |
|  | VRCDS | PPF-Grasp | E2E-GraspNet |
| 100 | 85 | 55 | 65 |
| 200 | 86 | 60 | 70 |
| 300 | 88 | 61 | 75 |
| 400 | 90 | 65 | 77 |
| 500 | 93 | 73 | 80 |
| 600 | 98 | 80 | 82 |
| Number of samples | Low speed conveyor belts power consumption | | |
|  | VRCDS | PPF-Grasp | E2E-GraspNet |
| 100 | 0.9 | 2.4 | 2.1 |
| 200 | 1.0 | 2.45 | 2.45 |
| 300 | 1.1 | 2.5 | 2.7 |
| 400 | 1.2 | 2.65 | 2.8 |
| 500 | 1.3 | 2.8 | 2.9 |
| 600 | 1.3 | 3.3 | 3.0 |

**The data in Figure 12**

| Number of samples | High speed conveyor belt efficiency | | |
| --- | --- | --- | --- |
|  | VRCDS | PPF-Grasp | E2E-GraspNet |
| 100 | 70 | 58 | 53 |
| 200 | 73 | 62 | 56 |
| 300 | 80 | 63 | 58 |
| 400 | 85 | 70 | 60 |
| 500 | 90 | 78 | 63 |
| 600 | 96 | 82 | 72 |
| Number of samples | High speed conveyor belt power consumption | | |
|  | VRCDS | PPF-Grasp | E2E-GraspNet |
| 100 | 0.6 | 1.9 | 1.6 |
| 200 | 0.8 | 2.2 | 1.7 |
| 300 | 1.0 | 2.4 | 1.8 |
| 400 | 1.1 | 2.6 | 2.0 |
| 500 | 1.3 | 2.8 | 2.3 |
| 600 | 1.3 | 3.2 | 2.7 |

**The data in Figure 13**

| Capture quantity | Low speed transfer capture average accuracy | | |
| --- | --- | --- | --- |
|  | VRCDS | PPF-Grasp | E2E-GraspNet |
| 100 | 91 | 72 | 82 |
| 200 | 92 | 75 | 84 |
| 300 | 92 | 78 | 86 |
| 400 | 91 | 75 | 83 |
| 500 | 89 | 74 | 85 |
| Capture quantity | Average accuracy of high-speed transmission capture | | |
|  | VRCDS | PPF-Grasp | E2E-GraspNet |
| 100 | 91 | 82 | 72 |
| 200 | 92 | 80 | 70 |
| 300 | 92 | 80 | 70 |
| 400 | 91 | 79 | 69 |
| 500 | 89 | 79 | 69 |

**The data in Figure 14**

| Time (day) | Low speed conveyor belts | | |
| --- | --- | --- | --- |
|  | VRCDS | PPF-Grasp | E2E-GraspNet |
| 1 | 97.82 | 84.83 | 90.07 |
| 2 | 95.32 | 85.62 | 87.54 |
| 3 | 94.86 | 85.62 | 86.53 |
| 4 | 94.86 | 82.35 | 85.56 |
| 5 | 93.52 | 78.23 | 84.63 |
| 6 | 93.52 | 78.52 | 83.56 |
| 7 | 92.86 | 78.22 | 82.55 |
| 8 | 92.11 | 78.12 | 81.63 |
| 9 | 91.57 | 77.69 | 80.37 |
| Time (day) | High speed conveyor belt | | |
|  | VRCDS | PPF-Grasp | E2E-GraspNet |
| 1 | 96.91 | 82.06 | 89.84 |
| 2 | 91.34 | 83.26 | 88.63 |
| 3 | 93.52 | 78.86 | 88.63 |
| 4 | 90.69 | 77.86 | 86.55 |
| 5 | 90.84 | 76.55 | 85.96 |
| 6 | 90.69 | 75.98 | 85.55 |
| 7 | 90.84 | 75.12 | 85.86 |
| 8 | 90.12 | 75.12 | 85.12 |
| 9 | 90.58 | 74.79 | 85.37 |
